# Histomorphological study of the spinal growth plates from the convex side and the concave side in adolescent idiopathic scoliosis

**DOI:** 10.1186/1749-799X-2-19

**Published:** 2007-11-11

**Authors:** Shoufeng Wang, Yong Qiu, Zezhang Zhu, Zhaolong Ma, Caiwei Xia, Feng Zhu

**Affiliations:** 1 Spine Surgery, Drum Tower Hospital, Nanjing University Medical School, Nanjing, China

## Abstract

Asymmetrical growth of the vertebrae has been implicated as one possible etiologic factor in the pathogenesis of adolescent idiopathic scoliosis. The longitudinal vertebral growth derives from the endochondral ossification of the vertebral growth plate. In the present study, the growth plates from the convex and concave side of the vertebrae were characterized by the method of histology and immunohistochemistry to evaluate the growth activity, cell proliferation, and apoptosis. Normal zoned architectures were observed in the convex side of the growth plate and disorganized architectures in the concave side. The histological grades were significantly different between the convex and the concave side of the growth plate in the apex vertebrae (P < 0.05). The histological difference was also found significant statistically between end vertebrae and apex vertebrae in the concave side of vertebral growth plates (P < 0.05). The proliferative potential indexes and apoptosis indexes of chondrocytes in the proliferative and hypertrophic zone in the convex side were significantly higher than that in the concave side in the apex vertebral growth plate (P < 0.05). There was a significant difference of the proliferative potential index (proliferating cell nuclear antigen, PCNA index) between convex side and concave side at the upper end vertebra (P < 0.05). The difference of the proliferative potential index and apoptosis index were found significant statistically in the concave side of the vertebral growth plate between end vertebrae and apex vertebrae (P < 0.05). The same result was also found for the apoptosis index (terminal deoxynucleotidyl transferase mediated deoxyuridine triphosphate biotin nick end labeling assay, TUNEL index) in the convex side of vertebral growth plate between end vertebrae and apex vertebrae (P < 0.05). Some correlation were found between radiographic measurements and proliferation and apoptosis indexes. The difference in histological grades and cellular activity between the convex and concave side indicated that the bilateral growth plate of the vertebrae in AIS patients have different growth kinetics which may affect the curve progression.

## Introduction

Adolescent idiopathic scoliosis (AIS) is a complex three-dimensional anomaly of the spine which involves lateral deviations on the frontal plane, misalignment on the sagittal plane, and spinal torsion. Asymmetric growth of the vertebrae was implicated as one possible etiologic factor in the pathogenesis of adolescent idiopathic scoliosis because the development and progression of scoliosis usually occurred during the rapid adolescent growth spurts [1-3]. Some research even reported that differential growth rates between the right and left side of the vertebrae could generate asymmetric growth and wedging of the vertebrae which may play an important role in the progression of the curve [4-8]. A large scale of scoliotic specimens was studied by Parent et al.[9]. They found that vertebral wedging was more prominent in the frontal plane, and there was minimal wedging in the sagittal plane. Whether the vertebral wedging in the frontal plane in AIS is the primary or the secondary change remains unclear. The clinical observation that the vertebral height on the concave side in the curve was smaller than that of the convex side makes us believe that vertebral asymmetric growth in the frontal plane plays a more important role in the progression of idiopathic scoliosis.

It was well known that the growth of the anterior column of vertebrae mainly came from the vertebral growth plate like the physes to the long bone which was important to the longitudinal vertebral growth [10-14]. The chondrocytes were regulated by the localized growth factors and the circulating systemic hormones to ensure a balance between the proliferation and apoptosis in the growth plate during the growth period [15-21]. Previous studies have showed that the activity of the chondrocytes in the growth plate was shown to be the indicators of the growth rate during the growth period [10-12,14]. To our knowledge, no studies were conducted to compare the difference of the growth activity and the proliferation and apoptosis of chondrocytes between the convex and concave side of the vertebral growth plate in AIS patients.

In the present study, cell proliferation and apoptosis can be specifically detected by the antibody against the proliferating cell nuclear antigen (PCNA), poly ADP ribose polymerase (PARP), and the terminal deoxynucleotidyl transferase mediated deoxyuridine triphosphate biotin nick end labeling assay (TUNEL) respectively. The proliferation and apoptosis indexes between the convex and concave side of the vertebral growth plate were compared. The proliferation and apoptosis indexes were correlated with radiographic measurements. The difference of growth activity between the convex side and the concave side of the vertebral growth plates was assessed by histological grading method.

## Materials and methods

### Clinical data and tissue sampling

From November 2004 to April 2006, the samples of vertebral growth plates were harvested from patients with idiopathic scoliosis who underwent anterior release and fusion for thoracic, lumbar, or thoracolumbar curves. Patients who suffered from congenital scoliosis, paralytic scoliosis, neuromuscular scoliosis, and the other types of scoliosis with known causes were excluded. A total of 21 female cases were included into this study. The study was approved by the University Ethics Committee. Consents were obtained from the patients and their parents. One hundred and twenty six vertebral growth plates were harvested from these patients. The patients were 11 to 18 years old (averaging 13.5 years old). Standing long cassette anteroposterior and lateral radiographs were taken and evaluated. The Cobb angle, apex vertical translation(AVT), apex vertebral rotation and disc wedging angle(DWA) of apex were measured. The curve types were classified according to the Lenke classification system[22] including three cases of Lenke 1A, five cases of 1B, seven of 1C, four cases of 5c, and two cases of 6C. The growth plates were dissected and retrieved from the apex and the upper and lower end vertebrae of the curve, and then were further separated into two groups: samples obtained from the concave side and the samples from the convex side. These growth plates were immediately fixed in 4% paraformaldehyde and transferred to the pathology department. After 24 hours, they were decalcified in 0.5 M ethylenediamine tetraacetic acid (EDTA) for two weeks. Subsequently, the specimens were fixed in paraffin wax. The embedded blocks were sectioned into 4–5 um slides and prepared for the staining of hematoxylin & eosin, immunohistochemistry, and in situ Cell Death Detection.

### Hematoxylin & eosin staining

**Figure 1:**
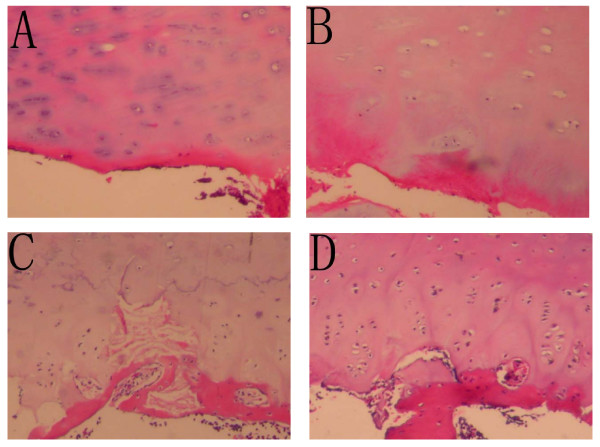
The histological grades of growth plates in adolescent idiopathic scoliosis. Grade 0 showing no signs of proliferative cartilage zone and growth activity(A). Grade I showing some proliferative cartilage zone but no growth activity(B). Grade II showing areas of growth inactivity and areas of proliferative cartilage zones(C). Grade III showing proliferative cartilage zones throughout the section(D).

All sections were stained with hematoxylin and eosin. The pathologic patterns of the vertebral growth plates were observed under the light microscope. The growth plates were graded histologically according to the grading system of vertebral endplates reported by Noordeen[20]. Grade 0 indicated no proliferative cartilage zone and no growth activity. Grade I showed no growth activity, but areas of proliferative cartilage zones were present. Grade II had areas of growth inactivity and areas of proliferative cartilage zones. Grade III indicated a proliferative cartilage zone throughout the section (Figure 1A–D). All the sections were assessed by two pathologists separately. According to Noordeen's specification, histological Grade 0 and Grade I were not considered to represent significant vertebral growth. Grade II and Grade III were regarded as active vertebral growth.

### *In situ *cell apoptosis detection

Detection of cell apoptosis was done by TUNEL assay (Roche, Mannheim, Germany) according to the manufacturer's protocol. In brief, 5 um tissue sections from paraffin-embedded growth plates were dewaxed in xylene, rehydrated, and pretreated with proteinase K (20 μg/mL in 100 mmol/L Tris pH 8.0/50 mmol/L edetic acid [EDTA]) for ten minutes at 37°C. Slides were rinsed twice in phosphate buffered saline (PBS) and incubated with TUNEL reaction mixture for one hour at 37°C in a humidified chamber. After washing with PBS 50 μL, Converter-AP solution was applied, and the slides were incubated for an additional 30 minutes at 37°C. The slides were washed again three times in PBS and incubated for ten minutes at ambient temperature after adding the chromogenic substrate FastRed (Roche). Slides were counterstained with hematoxylin, mounted under glass coverslips using Aquatex (Merck), and analyzed under a light microscope. As a negative control, the reaction was carried out without terminal transferase, and as a positive control, DNA strand breaks were induced by DNaseI treatment (Roche, 0.5 mg/mL).

### Immunohistochemistry

For immunohistochemical staining, monoclonal mouse antibodies (against PCNA protein, DAKO, Denmark) and affinity purified antibody from rabbit antiserum (against PARP LabVision, USA) were used. The paraffin sections were deparaffinized in xylene and rehydrated in graded alcohol (100%, 90%, 80%, and 70%). The endogenous peroxidase was subsequently blocked by 0.3% H_2_O_2 _for 30 minutes. After boiling in 10% citrate buffer (pH 6.0) for 15 minutes, the sections were incubated with relevant primary antibodies at 4°C for 16 hours. The sections were then exposed to a streptoavidinbiotin-peroxidase complex, and color was developed with 3, 3'-diaminobenzidine hydrochloride. Mayer's hematoxyline was used for counterstaining.

### Apoptosis (TUNEL positive, PARP positive) and proliferation potential (PCNA positive) indexes of chondrocytes

The total number of chondrocytes in the growth plate and the total number of apoptotic (TUNEL positive, PARP postive) and proliferative (PCNA positive) chondrocytes were counted twice in each sample (n = 21 for each group) with light microscopy. The percentage of TUNEL positive, PARP positive (apoptosis index), and PCNA positive (proliferation potential index) chondrocytes among the total number of chondrocytes in five random high power visual field (X40) was calculated on each side in one sample. The means of apoptosis and proliferation potential indexes were compared among the groups.

### Statistical test

SPSS version 10.0 (SPSS, Chicago) was used for statistical analysis. The values of different parameters were expressed as a mean with standard deviation. The means of proliferation indexes and apoptosis indexes were compared between the two sides of the growth plate with the paired sample t test. The difference of parameters between upper end, apex and lower end vertebrae were analyzed by one way analysis of variance. Fisher exact test was used for analyzing the difference of histological grades of the growth plates between convex side and concave side. Correlation of proliferation or apoptosis indexes and various radiographic measurements expressed as Pearson or spearmen correlation coefficients. P < 0.05 was considered significant.

## Results

### Histological grades

Each of the growth plates were first stained with hematoxylin and eosin. The zoned structure of the growth plate was observed in both the convex and concave side which could be divided into a resting, proliferative, hypertrophic and mineralized zone. The complete resting layer, proliferative layer, and hypertrophic layer were relatively shorter and clustered in the concave side.

The histological grades of the convex side were higher than that in the concave side in the apex, and significant difference was observed (P < 0.05). The histological difference was also found significant statistically between end vertebrae and apex vertebrae in the concave side of vertebral growth plates (P < 0.05) (Table 1).

**Table 1:** The difference of histological grades between the convex side and concave side of the vertebral growth plate

	Upper end		Apex		Lower end	
			
Locations HGs	0-I	II-III	0-I	II-III	0-I	II-III
Convex side	5	16	2	19*	4	17
Concave side	8	13^#^	19	2*^#+^	8	13^+^

Note: * indicates statistical significant between convex side and concave side(P < 0.05);^# ^indicates statistical significant between upper end vertebrae and apex vertebrae(P < 0.05); ^+^indicates statistical significant between lower end vertebra and apex vertebrae(P < 0.05)

### Proliferative potential indexes and apoptosis indexes

**Figure 2:**
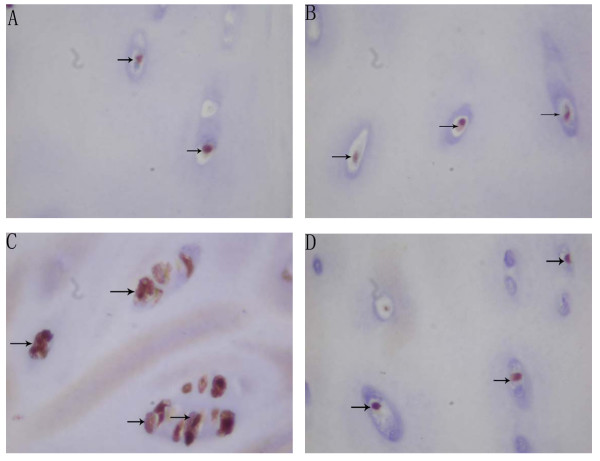
Microphotographs of PCNA-positive chondrocytes (arrows) in the resting zone and in the proliferative & hypertrophic zone of growth plate of apex vertebrae in AIS patient under micro camera(Magnification: 400×). PCNA-positive chondrocytes (arrows) in the resting zone of convex side(A). PCNA-positive chondrocytes (arrows) in the resting zone of concave side(B). PCNA-positive chondrocytes (arrows) in the proliferative & hypertrophic zone of convex side(C). PCNA-positive chondrocytes (arrows) in the proliferative & hypertrophic zone of concave side(D).

**Figure 3:**
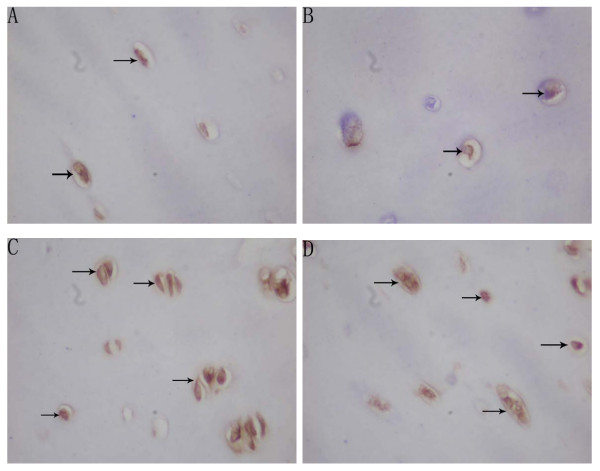
Microphotographs of TUNEL-positive chondrocytes in the resting zone and in the proliferative & hypertrophic zone of growth plate of apex vertebrae in AIS patient(Magnification: 400×). TUNEL-positive chondrocytes (arrows) in the resting zone of convex side(A). TUNEL-positive chondrocytes (arrows) in the resting zone of concave side(B). TUNEL-positive chondrocytes (arrows) in the proliferative & hypertrophic zone of convex side(C). TUNEL-positive chondrocytes (arrows) in the proliferative & hypertrophic zone of concave side(D).

**Figure 4:**
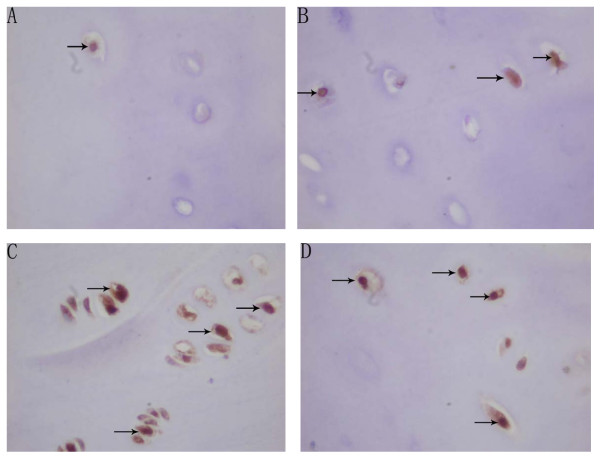
Microphotographs of PARP-positive chondrocytes in the resting zone and in the proliferative & hypertrophic zone of growth plate of apex vertebrae in AIS patient(Magnification: 400×). PARP-positive chondrocytes (arrows) in the resting zone of convex side(A). PARP-positive chondrocytes (arrows) in the resting zone of concave side(B). PARP-positive chondrocytes (arrows) in the proliferative & hypertrophic zone of convex side(C). PARP-positive chondrocytes (arrows) in the proliferative & hypertrophic zone of concave side(D).

There was no difference between the proliferation potential index and apoptosis index in the resting zone between the convex side and the concave side in each location (P > 0.05). Because of the indistinct separation between the proliferative and the hypertrophic zone in the concave side, the proliferative potential indexes and apoptosis indexes were evaluated through the proliferative and hypertrophic zone. The mean proliferative potential indexes (PCNA index) of chondrocytes in the proliferative and hypertrophic zone were 42.90% (SD, ± 11.46%) and 43.43% (SD, ± 5.47%) in the convex side of the growth plate of the upper end vertebrae and the apex vertebrae, which were higher than that (39.17%(SD, ± 5.13%), 25.63% (SD, ± 7.22%)) in the concave side in the same location, and there were statistical significance (P < 0.05). The mean proliferative potential indexes(PCNA index) of chondrocytes in the proliferative and hypertrophic zone in the concave side of the apex vertebral growth plate was lower than those in the upper and lower end vertebra(P < 0.05) (Table 2, Figure 2 A–D). The mean apoptosis indexes (TUNEL index) of chondrocytes in the proliferative and hypertrophic zone was 41.23% (SD ± 5.55%) in the convex side of growth plate of apex vertebrae, which was higher than that (26.13% (SD, ± 5.89%)) in the concave side in the same location, and there was a statistical significance (P < 0.05). The mean apoptosis indexes (TUNEL index) of chondrocytes in the proliferative and hypertrophic zone of the convex side of the upper and lower vertebral growth plates were found lower than those in the same side of apex vertebra (P < 0.05). However, in the contrast, the mean apoptosis indexes(TUNEL index) of chondrocytes in the proliferative and hypertrophic zone of the concave side of the upper and lower vertebral growth plates were found higher than those in the same side of apex vertebra (P < 0.05) (Table 3, Figure 3 A–D). The mean apoptosis indexes (PARP indexes) of chondrocytes in the proliferative and hypertrophic zone was 32.70% (SD, ± 6.45%) in the convex side the apex vertebral growth plate, which was higher than that (24.00% (SD, ± 7.24%)) in the concave side in the same location with statistical significance(P < 0.05) (Table 4, Figure 4). The same result was also found that the mean apoptosis indexes (PARP indexes) of chondrocytes in the proliferative and hypertrophic zone in the concave side of the upper and lower end vertebral growth plates were higher than that in the apex with statistical significance (P < 0.05) (Table 4, Figure 4 A–D). Some correlation were found between radiographic measurements and proliferation and apoptosis indexes (Table 5, 6).

**Table 2:** The proliferation potential indexes(PCNA indexes)(Mean ± SD) between the convex side and the concave side of the vertebral growth plate (%)

Locations		Upper end	Apex	Lower end
Resting zone	Convex side	2.78 ± 0.71	2.80 ± 0.71	2.69 ± 0.51
	Concave side	2.65 ± 0.56	2.77 ± 0.56	2.43 ± 0.76
Proliferative & Hypertrophic zone	Convex side	42.90 ± 11.46*	43.43 ± 5.47*	42.81 ± 3.10
	Concave side	39.17 ± 5.13*^#^	25.63 ± 7.22*^#+^	41.89 ± 3.27^+^

Note: * indicates statistical significant between convex side and concave side(P < 0.05);^# ^indicates statistical significant between upper end vertebrae and apex vertebrae(P < 0.05); ^+^indicates statistical significant between lower end vertebra and apex vertebrae(P < 0.05)

**Table 3:** The apoptosis indexes(TUNEL indexes) (Mean ± SD) between the convex side and the concave side of the vertebral growth plate (%)

Locations		Upper end	Apex	Lower end
Resting zone	Convex side	3.67 ± 0.89	3.89 ± 0.9	2.47 ± 0.39
	Concave side	3.46 ± 0.45	3.76 ± 0.4	2.56 ± 0.68
Proliferative & Hypertrophic zone	Convex side	36.09 ± 6.72^#^	41.23 ± 5.55*^#+^	36.67 ± 6.31^+^
	Concave side	33.82 ± 4.71^#^	26.13 ± 5.89*^#+^	35.70 ± 4.32^+^

Note: * indicates statistical significant between convex side and concave side(P < 0.05);^# ^indicates statistical significant between upper end vertebrae and apex vertebrae(P < 0.05); ^+^indicates statistical significant between lower end vertebra and apex vertebrae(P < 0.05)

**Table 4:** The apoptosis indexes(PARP indexes) (Mean ± SD) between the convex side and the concave side of the vertebral growth plate (%)

Locations		Upper end	Apex	Lower end
Resting zone	Convex side	2.45 ± 0.31	2.27 ± 0.39	2.67 ± 0.52
	Concave side	2.56 ± 0.6	2.41 ± 0.67	2.37 ± 0.35
Proliferative & Hypertrophic zone	Convex side	31.13 ± 6.79	32.70 ± 6.45*	31.69 ± 6.36
	Concave side	31.37 ± 4.26^#^	24.00 ± 7.24*^#+^	32.02 ± 6.02^+^

Note: * indicates statistical significant between convex side and concave side(P < 0.05);^# ^indicates statistical significant between upper end vertebrae and apex vertebrae(P < 0.05); ^+^indicates statistical significant between lower end vertebra and apex vertebrae(P < 0.05)

**Table 5:** Correlation of proliferation or apoptosis indexes to various radiographic measurements

Measurements			Cobb	AVT	AVR	DWA
Upper end vertebral growth plate	Convex side	PCNA	0.251	0.232	0.362	0.334
		TUNEL	0.184	0.090	0.204	0.166
		PARP	0.417	0.384	0.427	0.326
	Concave side	PCNA	-0.100	-0.099	-0.057	0.035
		TUNEL	-0.125	-0.202	-0.118	-0.005
		PARP	-0.488*	-0.549*	-0.295	-0.464*
Apex vertebral growth plate	Convex side	PCNA	0.453*	0.519*	0.498*	0.299
		TUNEL	0.395	0.324	0.493*	0.254
		PARP	0.563*	0.556*	0.641*	0.417
	Concave side	PCNA	-0.589*	-0.547*	-0.404	-0.538*
		TUNEL	-0.774*	-0.814*	-0.710*	-0.657*
		PARP	-0.339	-0.364	-0.185	-0.323
Lower end vertebral growth plate	Convex side	PCNA	-0.069	-0.024	0.266	0.106
		TUNEL	-0.080	-0.048	0.183	0.002
		PARP	-0.099	0.018	0.195	0.029
	Concave side	PCNA	-0.106	-0.046	0.166	-0.010
		TUNEL	-0.240	-0.224	-0.232	-0.146
		PARP	-0.275	-0.179	0.080	-0.170

Correlation of proliferation or apoptosis indexes with various radiographic measurements expressed as Pearson or spearmen correlation coefficients with significance set at P < 0.05. * Statistical significance.

AVT = Apex vertical translation; AVR = Apex vertebral rotation; DWA = Disc wedging angle of apex.

**Table 6:** Correlation of difference of proliferation or apoptosis indexes between convex and concave side to various radiographic measurements

Measurements			Cobb	AVT	AVR	CWAD
Upper end vertebral growth plate	Convex-Concave	PCNA	0.646*	0.607*	0.573*	0.612*
		TUNEL	0.603*	0.557*	0.541*	0.341
		PARP	0.717*	0.729*	0.679*	0.618*
Apex vertebral growth plate	Convex-Concave	PCNA	0.825*	0.845*	0.662*	0.662*
		TUNEL	0.898*	0.900*	0.850*	0.722*
		PARP	0.756*	0.776*	0.505*	0.632*
Lower end vertebral growth plate	Convex-Concave	PCNA	0.055	0.037	0.153	0.237
		TUNEL	0.228	0.256	0.516*	0.223
		PARP	0.229	0.264	0.581*	0.269

Correlation of difference of proliferation or apoptosis indexes between convex and concave side with various radiographic measurements expressed as Pearson or Spearmen correlation coefficients with significance set at P < 0.05. * Statistical significance.

AVT = Apex vertical translation; AVR = Apex vertebral rotation; DWA = Disc wedging angle of apex.

## Discussion

The role of spinal growth on the development and progression of AIS was already well described in literature [23-26]. Unbalanced growth between the right and left side of the spine which could induce spinal asymmetry was reported [4-7]. This asymmetric growth may leads to the progression of deformity. Dickson et al. [27] suggested that idiopathic scoliosis presented asymmetry of the spine in both the coronal and the sagittal plane which was an essential characteristic of idiopathic scoliosis. Stilwell [28] and Michelsson[29] speculated that the main pathogenesis of scoliosis was asymmetrical bone growth. Histologic studies were performed on the cartilaginous growth plate by them in the vertebrae of animals with scoliosis. Decreased chondrogenesis, disorganized columnation, and premature cessation of growth in the cartilaginous growth plate of the vertebral body were observed [28,29]. In human beings, McCarroll and Costen[30] obtained biopsies of the lateral aspect of the thoracic vertebral cartilaginous growth plates on the convex side of the curve in the course of performing unilateral growth-arrest operations in idiopathic scoliosis. These biopsies showed confusion and retardation of cartilaginous growth. In the present study, it is demonstrated that proliferative zone and hypertrophic zone of the growth plate were more compact and clustered together which was different from long bone growth plate illustrated in text books. There was a significant difference of histological grades between the convex side and the concave side in the upper end, the apex, and the lower end vertebrae. The proliferative potential indexes and apoptosis indexes indicate that a distinct difference of proliferation and apoptosis of chondrocytes exists between the convex side and concave side of the growth plate at the apex.

In our study, a significant difference of histological grades between two sides at the apex of the curve indicates that a significant difference of growth activity between the convex side and concave side of the growth plate may exist. The different growth activity of growth plates may affect the bone formation and vertebral growth in coronal plane subsequently which may plays an important role in the progression of AIS.

Increases in the lengths of long bones and the heights of vertebrae are generated by proliferation of the growth plate chondrocytes, their enlargement in the growth direction, and the synthesis of the matrix that eventually calcifies [10-14,31,32]. Wilsman et al. [12] studied the four different growth plates in 28-day-old Long-Evans rats and found that the number of new chondrocytes produced per day varied in the different growth plates and correlated positively with the rate of elongation, but these studies all were conducted through the animal model. The proliferation and apoptosis of vertebral growth plate chondrocytes were rarely studied in human beings with scoliosis. In our study, the percentage of the PCNA positive chondrocytes in the proliferative and the hypertrophic zone in the convex side of the growth plate was higher than that in the concave side in the apex vertebrae. This implicates that there may exist a different proliferative activity of the chondrocytes between two sides of the apex growth plate. The similar results were found in the apoptosis indexes. Most of the apoptotic chondrocytes appeared in the hypertrophic zone and mineralization zone. However, most of the proliferative potential indexes and apoptosis indexes were not found statistically significant between convex and concave side of the end vertebral growth plates except for the PCNA indexes in the upper end vertebra. Some correlations(positive or negative) were found between proliferation or apoptosis indexes and radiographic measurements. The difference of proliferation or apoptosis indexes between convex and concave side correlated mostly with various radiographic measurements in the upper end and apex vertebral growth plate. These finding implicated that the vertebral growth plates may be affected by a mechanical cause point to the Hueter-Volkmann law, which states that growth is retarded by mechanical compression and accelerated by distraction or reduced compression of the growth plate relative to normal values [2,32]. It is important to recall that the scoliotic tissue we analyzed mainly represents the convex and concave side of the entire scoliotic tissue (growth plates), in which the tissue is experiencing tension or compression. The difference of proliferative potential indexes and apoptosis indexes in the concave side between the end and apex vertebral growth plates may also be a result affected by different mechanical conditions. In the previous study, proliferation and apoptosis of chondrocytes must coordinate well together and ensure the normal endochondral bone formation and longitudinal bone growth subsequently [10-12]. In our study, the differential proliferation indexes and apoptosis indexes of chondrocytes between the convex side and the concave side of the vertebral growth plate implicates that a different chondrocytic kinetics may exist and contribute to the differential growth rate between two sides of the vertebrae which will be followed by the differential growth between two sides of the vertebrae in the coronal plane and the wedging of the vertebrae at the apex. Therefore, these findings may be secondary to the changes of different mechanical conditions, but which may indeed play an important role in the curve progression.

Although whether the wedging of the vertebrae in the coronal plane being the primary cause or secondary change was unclear, differential growth between the right and left side of the vertebrae could generate asymmetry may indeed involve in the progression of AIS [33-35]. The study may provide some histological cues to the progression of the curve. But, the vertebral growth is a complex progress. The modulation of vertebral endochondral bone formation, like long bones, is controlled by local factors and systemic factors [36-40]. Further studies should focus on the matrix synthesis and local and systemic factors to understand the underlying mechanism that causes the difference.

The limitations of the present study were that, firstly, there is no control group from non-scoliotic patients; secondly, it should be noted that, during the operation, the growth plate from the concave side were obtained as far from the midline of the vertebral body as possible. But in order not to injure the aorta, the growth plate from the concave side was not the absolute concave side of the growth plate. Thirdly, because of the difficulty for the acquirement of sample at the end vertebrae during surgery, some of the vertebral growth plates may be not the real growth plates of the end vertebrae. The last one is the different cell density between convex and concave side of growth plates. In the severe curves or the apex, the cell density may be very low especially in the concave side. The percentage of positive chondrocytes as the proliferation or apoptosis index may offset the impact of low cell density.

## Conclusion

The difference in histological grades and cellular activity between the convex and concave side indicated that the bilateral growth plate of the vertebrae in AIS patients have different growth kinetics which may affect the curve progression.

## Competing interests

The author(s) declare that they have no competing interests. No benefits in any form have been received or will be received from a commercial party related directly or indirectly to the subject of this article.

## Authors' contributions

The first three authors contributed to the planning, execution and completion of the project. The article was written up by the first author with advice and guidance from the second (senior) author who conceptualized the topic of this article. All authors read and approved the manuscript.
